# Iron-Induced Changes in the Proteome of *Trichomonas vaginalis* Hydrogenosomes

**DOI:** 10.1371/journal.pone.0065148

**Published:** 2013-05-31

**Authors:** Neritza Campo Beltrán, Lenka Horváthová, Petr L. Jedelský, Miroslava Šedinová, Petr Rada, Michaela Marcinčiková, Ivan Hrdý, Jan Tachezy

**Affiliations:** Department of Parasitology, Charles University in Prague, Faculty of Science, Prague, Czech Republic; The Scripps Research Institute and Sorrento Therapeutics, Inc., United States of America

## Abstract

Iron plays a crucial role in metabolism as a key component of catalytic and redox cofactors, such as heme or iron-sulfur clusters in enzymes and electron-transporting or regulatory proteins. Limitation of iron availability by the host is also one of the mechanisms involved in immunity. Pathogens must regulate their protein expression according to the iron concentration in their environment and optimize their metabolic pathways in cases of limitation through the availability of respective cofactors. *Trichomonas vaginalis*, a sexually transmitted pathogen of humans, requires high iron levels for optimal growth. It is an anaerobe that possesses hydrogenosomes, mitochondrion-related organelles that harbor pathways of energy metabolism and iron-sulfur cluster assembly. We analyzed the proteomes of hydrogenosomes obtained from cells cultivated under iron-rich and iron-deficient conditions employing two-dimensional peptide separation combining IEF and nano-HPLC with quantitative MALDI-MS/MS. We identified 179 proteins, of which 58 were differentially expressed. Iron deficiency led to the upregulation of proteins involved in iron-sulfur cluster assembly and the downregulation of enzymes involved in carbohydrate metabolism. Interestingly, iron affected the expression of only some of multiple protein paralogues, whereas the expression of others was iron independent. This finding indicates a stringent regulation of differentially expressed multiple gene copies in response to changes in the availability of exogenous iron.

## Introduction

Iron is an essential element for virtually all forms of life. It plays an indispensable role as a component of metalloproteins, either bound to more or less complex prosthetic groups, such as heme or iron-sulfur (FeS) clusters, or alone, such as in the case of ribonucleotide reductase, the key enzyme of DNA metabolism. Metalloproteins are involved in many vital cellular functions, including electron transport, enzymatic catalysis, redox sensing and regulation of gene expression [1]. In addition to housekeeping functions, iron influences the virulence of pathogenic microorganisms, which is underlined by the observations that host iron withholding is markedly reinforced during microbial infection [2]. Therefore, invading pathogens have evolved effective iron-acquisition mechanisms to meet their needs for iron [3].

*Trichomonas vaginalis* is a sexually transmitted anaerobic parasitic protist of the Excavata group that infects humans, with an estimated worldwide annual incidence of 170 million cases [4], [5]. One of the most prominent characteristics of this parasite is the lack of “classical” oxygen-respiring mitochondria. Instead, trichomonads possess hydrogenosomes, mitochondria-type organelles that produce molecular hydrogen and among other functions, synthesize ATP through substrate-level phosphorylation [6]. Trichomonads require unusually high concentrations of iron in in vitro cultures [7]. This need has been largely attributed to the dependence of trichomonads upon the activities of FeS cluster-containing proteins, which mediate vital energy-conserving reactions in the parasite’s hydrogenosomes [8], but it may also be related to the fact that trichomonads apparently lack substantial levels of iron-storage proteins, such as ferritin [3]. Thus, high extracellular iron may be required to furnish the turnover of FeS proteins. Lactoferrin, heme and low-molecular-weight iron complexes can serve as an external source of iron for *T. vaginalis* [9].

The FeS proteins involved in hydrogenosomal energy metabolism are pyruvate:ferredoxin oxidoreductase (PFO), electron carrier ferredoxin, [FeFe]-hydrogenase and two-subunit remnants of respiratory complex I. PFO oxidatively decarboxylates pyruvate, which is supplied from the cytosol or through the activity of non-FeS hydrogenosomal malic enzyme, to acetyl-CoA, which is converted to acetate by the activity of acetate:succinate-CoA transferase in a succinate-dependent reaction. The resulting succinyl-CoA serves as a substrate for ATP synthesis by succinate thiokinase, while electrons released from pyruvate are transported via ferredoxin to hydrogenase, which forms molecular hydrogen (see [9] for review). NADH resulting from the malic enzyme reaction can be reoxidized by the complex I remnant [10]. In addition to the proteins involved in hydrogenosomal carbohydrate metabolism, other hydrogenosomal Fe-containing proteins include FeS flavoproteins, the flavodiiron oxygen reductase, peroxidase rubrerythrin, proteins of the FeS cluster assembly system (ISC), hybrid cluster protein, Fe superoxide dismutase and possibly others [11]–[14].

Iron availability markedly influences hydrogenosomal catabolism, and increased activities of FeS enzymes involved in hydrogenosomal energy metabolism have been observed upon iron supplementation [7], [8], [15]. Clearly, there is effective regulation linking the activity/expression of FeS proteins and iron availability. Even the expression of non-FeS proteins, such as malic enzyme, has been shown to be regulated by iron [8], [15]–[16]. The list of iron-regulated genes has been greatly extended by our previous work, which demonstrated a marked effect of iron on the *T. vaginalis* transcriptome [18]. Among the hundreds of regulated genes identified in this study, hydrogenosomal carbohydrate metabolism and ISC assembly machinery appeared to be the most important pathways influenced by this critical nutrient. *T. vaginalis* genes are typically present in multiple copies [19], and intriguingly, in most cases expression of only certain paralogues was regulated by iron [18]. The effect of iron limitation on *T. vaginalis* morphology and overall proteome changes was studied by De Jesus et al. [20]. Cells from iron-depleted medium displayed altered morphology, including the internalization of flagella and the axostyle and transformation to a larger and rounded shape. Observed changes in protein expression included the downregulation of PFO and cysteine proteases, while actin was upregulated in iron-depleted trichomonads [21]. However, the two-dimensional gel electrophoresis (2DE) used to separate the protein samples prior to MS identifications in the previous study is known to often fail to resolve membrane proteins, low-abundance proteins, proteins with extreme pI values and very small or large proteins [22], resulting in an incomplete list of identified proteins, potentially including those affected by changes in external conditions. Therefore, to obtain a better picture of the effect of iron limitation on trichomonads, we utilized a gel-free approach based on isobaric tag labeling (iTRAQ), isoelectric focusing of tryptic peptides and nano-LC-MALDI identification. We specifically focused on hydrogenosomes because many FeS proteins reside in these organelles, along with the FeS cluster assembly machinery.

## Methods

### Parasite Cultivation

*Trichomonas vaginalis* strain T1 (J.H. Tai, Institute of Biomedical Sciences, Taipei, Taiwan) was grown in Diamond’s trypticase-yeast-extract-maltose (TYM medium) supplemented with 10% heat-inactivated horse serum without agar at pH 6.2 [23]. The iron-supplemented medium was prepared by adding ammonium ferric citrate to a final iron concentration of 86 µM. Iron-restricted cells were subcultured for 10 passages in iron-deficient TYM medium prepared without ammonium ferric citrate and supplemented with 2,2′-dipyridyl (Sigma Chemical Co., St. Louis, Missouri) to a final concentration of 70 µM.

### Cell Fractionation and Hydrogenosome Isolation

One-liter cultures of *T. vaginalis* cells grown under iron-enriched (+Fe) and iron-depleted (−Fe) conditions were harvested by centrifugation at 1300×g for 12 minutes at 4°C and washed twice with 50 ml of phosphate-buffered saline (PBS) and once with 50 ml of isotonic ST buffer (250 mM sucrose, 10 mM Tris, and 0.5 mM KCl, pH 7.2). Subsequent steps were performed at 4°C in ST buffer supplemented with the protease inhibitors TLCK 50 µg/ml (tosyl lysyl chloromethyl ketone) and leupeptin 10 µg/ml. Cell pellets were resuspended in 40 ml of ST buffer and sonicated on ice until approximately 90% of the cells were disrupted. The homogenate was centrifuged at 800×g for 15 minutes to remove the nuclei and unbroken cells. The supernatant was centrifuged at 17000×g for 20 minutes, resulting in an enriched large granular fraction (LGF, sediment) and crude cytosolic fraction (supernatant). The enriched LGF was further fractionated using a discontinuous Optiprep^tm^ gradient (Axis-Shield). To prepare the gradient, 0.5 ml of 50% (w/v) Optiprep working solution was applied to the bottom of the tube, and 1 ml of each lower-density solution (ranging from 36 to 18% in 2% steps) was layered successively. The Optiprep working solution was prepared by diluting the original Optiprep^tm^ to 50% using a diluent recommended for general purposes (0.25 M sucrose, 6 mM EDTA and 60 mM Tris-HCl, pH 7.4). Successive gradient solutions were prepared by diluting the 50% working solution with homogenization buffer (0.25 M sucrose, 1 mM EDTA and 10 mM Tris-HCl, pH 7.4) to the specific concentration. The LGF was resuspended in 0.5 ml of homogenization buffer and layered on top of the gradient. The gradient was centrifuged in a swinging bucket rotor at 200,000×g for 2 hours at 4°C. The separated fractions were then carefully removed using a micropipette, and each fraction was washed separately with ST buffer containing protease inhibitors at 21,000×g for 20 minutes at 4°C.

### SDS-PAGE and Western Blotting

SDS-PAGE and Western blotting were used to analyze the protein composition of Optiprep-sucrose-purified hydrogenosomes. SDS-PAGE was performed with a Bio-Rad miniprotean gel apparatus using a 12% gel. Electrophoretically resolved proteins were stained with Coomassie brilliant blue or transferred to a nitrocellulose membrane to be probed with polyclonal rabbit antiserum against hydrogenosomal malic enzyme [24].

### Transmission Electron Microscopy (TEM)

Pellets of each fraction obtained from the Optiprep-sucrose gradients were fixed for 24 hours in 2.5% glutaraldehyde in 0.1 M cacodylate buffer (pH 7.2) and postfixed in 2% OsO_4_ in the same buffer. Fixed specimens were dehydrated with an ascending ethanol and acetone series and embedded in an Araldite - Poly/Bed® 812 resin mixture. Thin sections were cut on a Reichert-Jung Ultracut E ultramicrotome and stained using uranyl acetate and lead citrate. Sections were examined and photographed using a JEOL JEM-1011 electron microscope with a Megaview III camera and analySIS 3.2 software (Soft Imaging System®).

### Determination of Enzymatic Activities

The activities of hydrogenosomal and non-hydrogenosomal enzymes were measured spectrophotometrically at 25°C in all fractions. Hydrogenosomal malic enzyme was measured aerobically at 340 nm as the rate of malate-dependent NAD^+^ reduction [24], and the lysosomal marker enzyme acid phosphatase was measured according to Barret (1972). The activities were determined immediately after organelle isolation. Protein concentrations in the fractions were determined by the Lowry method.

### Protein Digestion and iTRAQ Labeling

Aliquots of hydrogenosomal fractions containing 100 µg of total protein were precipitated with 300 µl of acetone overnight at −20°C. The precipitate was centrifuged, acetone was carefully removed, and the remaining traces of acetone were left to evaporate for 5 minutes. Sample dissolution, reduction, alkylation, digestion and iTRAQ 4-plex labeling were performed according to the manufacturer’s instructions (AB Sciex, Foster City, CA) using sequencing-grade porcine trypsin (Promega). Combined samples were precipitated with 500 µl of acetone overnight at −20°C.

### Isoelectric Focusing, Extraction and HPLC

Combined iTRAQ-labeled samples were dissolved in 250 µl of 2 M urea and poured into the 17 cm focusing tray of a Protean IEF Cell (Bio-Rad, Hercules, CA, USA). The sample was covered with 17 cm IPG strips (pH 3–10, Bio-Rad) without paper wicks or oil. Active rehydration at 50 V for 2 hours was followed by voltage steps of 100, 250, 500 and 1000 V for 15 minutes and a maximum of 10 kV until 40 kVHrs was reached. The final step was set at 500 V forever. The current was limited to 50 µA, and only one strip was focused at a time.

The strip was cut into pieces approximately 2–3 mm wide. The pieces were sonicated for 15 minutes in 20 µl of 10% acetonitrile (ACN) with 0.1% trifluoroacetic acid. The supernatants were mixed 1∶1 with water and subjected to nano-reverse-phase HPLC. LC separation was performed with an Ultimate 3000 HPLC system (Dionex, Framingham, MA) coupled to a Probot micro-fraction collector (Dionex). A PepMap 100 C18 RP column (particle size 3 µm, length 15 cm, internal diameter 75 µm; Dionex) with pre-column (PepMap 300 C18, particle size 5 µm, 300 Å wide pore, length 5 mm, internal diameter 300) was used for separation with a gradient of 4% (v/v) acetonitrile and 0.1% (v/v) trifluoroacetic acid to 80% (v/v) acetonitrile and 0.1% (v/v) trifluoroacetic acid for 60 minutes. The flow rate was set to 300 nl/min. The eluate was mixed 1∶3 with matrix solution (2 mg/ml α-cyano-4-hydroxycinnamic acid in 80% ACN) with the Probot micro-fraction spotter prior to spotting onto a MALDI target. The spotting frequency was 5 spots per minute; i.e., 60 nl eluate +180 nl matrix solution per MALDI spot.

### Mass Spectrometry

Spectra were acquired on a 4800 Plus MALDI TOF/TOF analyzer (AB Sciex) equipped with an Nd:YAG laser (355 nm, firing rate 200 Hz). All spots were first measured in MS mode from m/z 800 to 4,000, and then, up to the 15 strongest precursors were selected for MS/MS analysis, which was performed with 1 kV collision energy and the operating pressure of the collision cell set to 10^−6^ Torr. Tandem mass spectra were processed with a 4000 Series Explorer with subtract baseline enabled (peak width 50), Gaussian smoothing applied with filter width 5, minimum signal to noise 8, local noise window width 250 m/z, minimum peak width at full width half max 2.9 bins, cluster area signal to noise optimization enabled (threshold 15) and flag monoisotopic peaks enabled (generic formula C_6_H_5_NO).

### Proteomic Data Analysis

A database search was performed with GPS Explorer v. 3.6 (AB Sciex) with locally installed Mascot v. 2.1 (Matrix Science) against the database of annotated *T. vaginalis* protein sequences from TrichDB (http://trichdb.org, release-1.2, 21-Sep-2010, 119344 sequences) with trypsin digestion, methyl methanethiosulfonate modification of cysteines and N-terminal and an ε-amino group of lysine modified with iTRAQ 4-plex reagents as fixed modifications and methionine oxidation as the variable modification. Precursor tolerance was set to 100 ppm, and the MS/MS fragment tolerance was 0.2 Da. The maximum peptide rank was 1, and the minimum ion score confidence interval (CI) per peptide was 95%. Spectra assigned to more than one protein were not used for quantitation. Average iTRAQ ratios and standard deviations were calculated for each protein using all of the available treatment/control iTRAQ pairs.

Bioinformatic searches based on protein BLAST (http://www.ncbi.nlm.nih.gov/blast) and hidden Markov models (http://toolkit.tuebingen.mpg.de/hhpred) were used to verify and manually edit TrichDB annotations. All identified protein sequences were analyzed using programs for subcellular localization prediction, including PSORT II (http://psort.hgc.jp/form2.html), TargetP (http://www.cbs.dtu.dk/services/TargetP/), Euk-mPLoc 2.0 (http://www.csbio.sjtu.edu.cn/bioinf/euk-multi-2/) and Yloc (http://abi.inf.uni-tuebingen.de/Services/YLoc/webloc.cgi). The NetBeans Platform application (Hunter software) was used to predict hydrogenosomal N-terminal targeting sequences as described previously [25].

## Results and Discussion

### Identification of Hydrogenosomal Proteins by Mass Spectrometry

**Figure 1:**
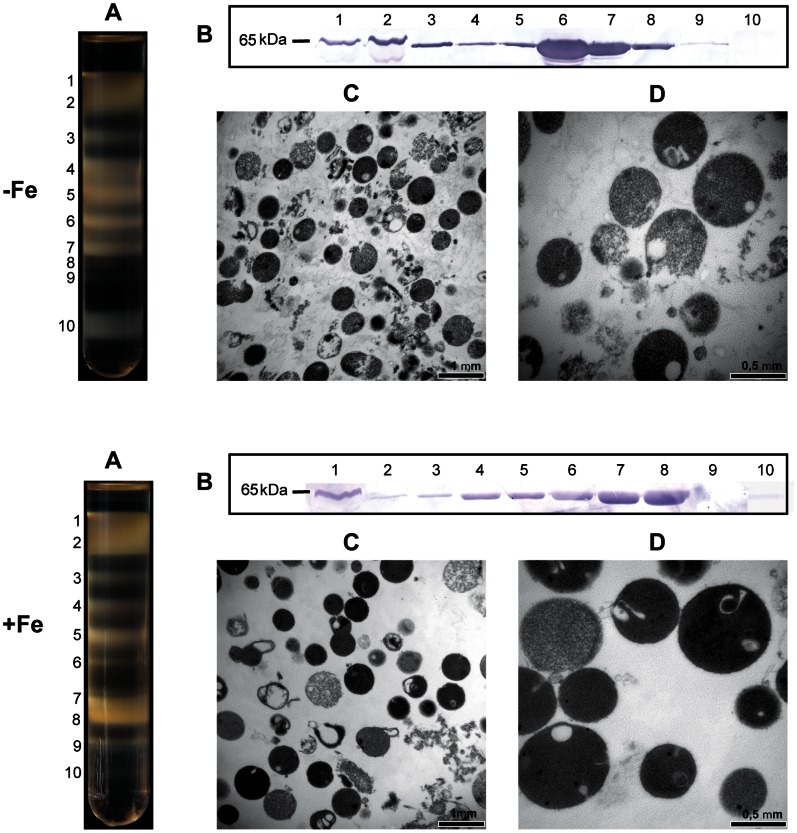
Isolation of hydrogenosomal fractions from *T. vaginalis* cells grown under iron-enriched (+Fe) and iron-depleted (−Fe) conditions. (A) The fraction enriched in hydrogenosomes was further fractionated with an Optiprep density gradient. (B) Analysis of Optiprep fractions by Western blotting using an antibody against hydrogenosomal malic enzyme. (C–D) Transmission electron microscopy of fraction #7, which was chosen for proteomic analysis.

To investigate changes in the *T. vaginalis* hydrogenosomal proteome caused by iron limitation, trichomonads were grown in media supplemented with 70 µM of the iron chelator 2,2-dipyridyl (iron-restricted conditions, −Fe). As a control, we used trichomonads grown in media supplemented with ammonium ferric citrate to a final iron concentration of 86 µM (iron-rich conditions, +Fe). Highly purified hydrogenosomes were obtained from homogenates of both cultures through differential centrifugation followed by preparative centrifugation of the large granular fraction using a discontinuous Optiprep (iodixanol) gradient. Ten distinct bands with variable thickness and density were obtained under both iron conditions. The band appearance/distribution differed between the +Fe and -Fe conditions (Fig. 1). Western blot analyses of fractions showed that the hydrogenosomal marker malic enzyme was particularly enriched in fractions #7 and #8 under iron-rich (+Fe) conditions and fractions #6 and #7 under iron-depleted (−Fe) conditions (Fig. 1). The fraction purity was further examined with electron microscopy (Fig. 1) and the determination of enzymatic activities of the hydrogenosomal and lysosomal markers malic enzyme and acid phosphatase, respectively (Fig. S1). Based on these results, fraction #7 appeared to be the purest hydrogenosomal fraction with the least contamination and was therefore chosen for the comparative proteomic analysis.

Proteins in this fraction were digested and labeled using the iTRAQ 4-plex kit. The labeled peptides were fractionated using isoelectric focusing and analyzed using LC-MS/MS. Five independent biological replicates were included in the analysis. Two pairs of biological replicates were processed and measured in two iTRAQ -LC-MS analyses, and one pair was measured in a separate analysis. In total, we acquired over 64,000 MS/MS spectra in 200 LC runs. The Mascot 2.1 search engine identified a total of 631 proteins, which were then classified into functional categories (Table S1). Spectra assigned to more than one protein were not used for quantitation. The average ratio for each protein was calculated from all available iTRAQ pairs. Only values calculated using at least three pairs were included. Proteins with an average ratio (fold change) of at least ±2.0 were considered differentially expressed.

To distinguish hydrogenosomal proteins from non-hydrogenosomal contamination, five different bioinformatic tools for the prediction of subcellular localization (PSORT II, TargetP, Euk-mPLoc 2.0, and Yloc and Hunter) were used (Table S1). A protein was considered putatively hydrogenosomal if mitochondrial localization was predicted by at least one of the tools. These predictions yielded 287 putative hydrogenosomal proteins (Table S1). In the final list of hydrogenosomal proteins (Table S2), we excluded all proteins in categories that were not related to the hydrogenosome and were most likely externally associated with other organelles (cytoskeleton, histone/DNA, vacuolar proteins, protein synthesis and modification, signaling pathways, signal transduction and vesicle transport) [14] and proteins for which non-hydrogenosomal localization was confirmed experimentally [13], [26]. However, we included proteins and their paralogues that were not recognized by the above-mentioned tools but were experimentally identified as hydrogenosomal proteins by others [13], [14], [26]. Using this approach, we selected 179 proteins (Table S2). We used relatively stringent criteria to eliminate all probable contaminants, and therefore, the final number of putative genuine hydrogenosomal proteins was considerably lower than that previously published by Schneider et al. [14]. Nevertheless, we identified several new paralogues of known hydrogenosomal proteins (e.g., ferredoxins, Nfu and Isd11) and proteins that had been overlooked by previous analyses [13], [14], such as frataxin and HydE (Table S2). Altogether, of the 179 proteins identified as likely to be hydrogenosomal, we obtained a fold change of at least 2.0 for 58 proteins; 31 of these proteins were upregulated, and 27 were downregulated under iron-deficient conditions (Table 1).

**Table 1:** Significantly regulated proteins in iron depleted conditions.

DOWNREGULATED			UPREGULATED		
Accession No.	Annotation	Fold change	Accession No.	Annotation	Fold change
TVAG_230580	Pyruvate:ferredoxin oxidoreductase BI	−37,4	TVAG_469020	HydG-1	10,4
TVAG_254890	Pyruvate:ferredoxin oxidoreductase E	−16,9	TVAG_146780	Nfu-4	8,7
TVAG_242960	Pyruvate:ferredoxin oxidoreductase BII	−13,5	TVAG_479680	2-nitropropane dioxygenase precursor	5,4
TVAG_310050	[Fe] hydrogenase-3	−9,0	TVAG_451860	Nfu-3	4,7
TVAG_265760	FAD/FMN-binding family protein-4	−8,8	TVAG_253630	Hsp70 mitochondrial type-3	3,5
TVAG_292710	Ferredoxin 4	−6,6	TVAG_008840	Nfu-2	3,2
TVAG_064490	Rubrerythrin-1	−6,4	TVAG_055320	IscA2-2	3,2
TVAG_154730	Iron-sulfur flavoprotein (ISF3)	−6,3	TVAG_044500	Nfu-1.	3,1
TVAG_399860	Ferredoxin 2	−5,9	TVAG_361540	IscA2-3	2,8
TVAG_198110	Pyruvate:ferredoxin oxidoreductase A	−5,4	TVAG_329200	HydE-2	2,7
TVAG_049140	Superoxide dismutase [fe], putative	−5,3	TVAG_282580	Conserved unknown protein	2,6
TVAG_183790	Malic enzyme F	−4,8	TVAG_412560	OsmC-2	2,5
TVAG_076510	Serine palmitoyltransferase	−4,2	TVAG_048590	Thioesterase family protein	2,5
TVAG_412220	Malic enzyme D.	−3,8	TVAG_344280	Conserved unknown protein	2,5
TVAG_296220	Complex 1, Tvh21	−3,6	TVAG_342900	Flavine reductase	2,4
TVAG_037570	[Fe] hydrogenase-2 (64 kDa)	−3,3	TVAG_060450	Acetyltransferase-1	2,3
TVAG_395550	Acetate:succinate CoA transferase-3	−3,2	TVAG_385350	Thioredoxin	2,3
TVAG_466790	Pyruvate:ferredoxin oxidoreductase F	−3,0	TVAG_456770	IscA2-1	2,3
TVAG_181350	Conserved unknown protein	−2,9	TVAG_242760	Isd11-1	2,2
TVAG_133030	Complex 1, Tvh47	−2,9	TVAG_182340	Mge (GrpE) protein −1	2,2
TVAG_003900	Ferredoxin 1	−2,8	TVAG_183850	Arginine deiminase −3	2,1
TVAG_416100	Malic enzyme C	−2,4	TVAG_370860	Tim17/22/23C	2,1
TVAG_047890	Succinyl-CoA synthetase, alpha	−2,3	TVAG_177600	Glycine cleavage system H protein	2,1
TVAG_182620	[Fe] Hydrogenase-1 (50 kDa)	−2,3	TVAG_381290	Hsp20-3	2,0
TVAG_340290	Malic enzyme H	−2,1	TVAG_277380	Ind-4 (P-Loop ATPase)	2,0
TVAG_104250	Hmp35 -2	−2,1	TVAG_205390	HydF	2,0
TVAG_351540	FAD/FMN-binding family protein-3	−2,0	TVAG_086470	Thioredoxin	2,0
			TVAG_433130	Hsp70 mitochondrial type-6	2,0
			TVAG_467820	Arginine deiminase -1	2,0
			TVAG_318670	Succinyl-CoA synthetase, alpha	2,0
			TVAG_109540	Serine hydroxymethyltransferase	2,0

### Iron-sulfur Cluster Assembly

**Figure 2:**
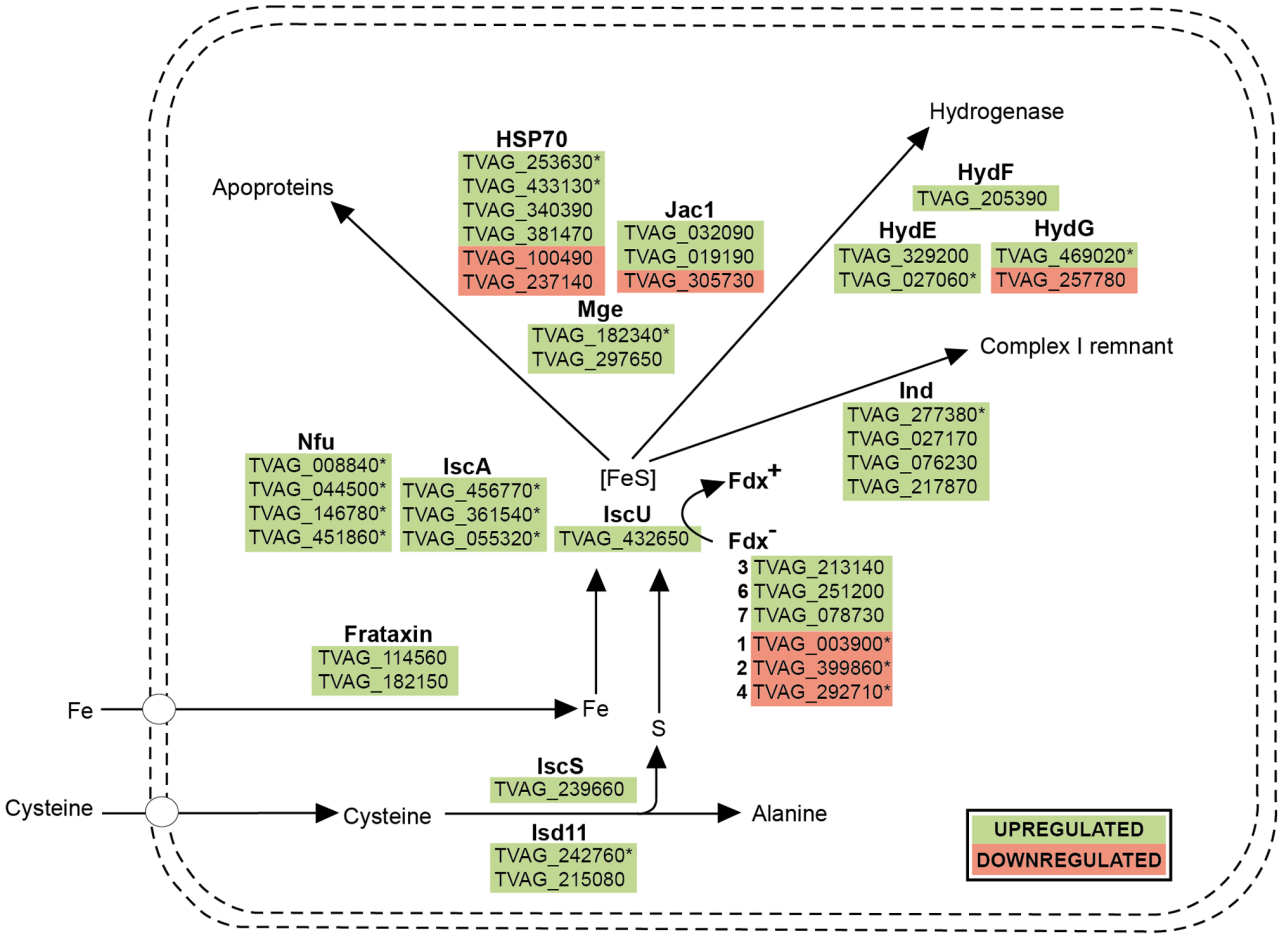
Proteins of the iron-sulfur cluster assembly machinery identified in this study. Gene IDs marked with an asterisk denote proteins that were significantly regulated in response to iron availability.

Hydrogenosomes of *T. vaginalis* possess machinery required for the formation of FeS clusters, which is homologous to the mitochondrial ISC system [27]–[29]. Iron depletion caused the increased expression of almost all known components involved in hydrogenosomal ISC assembly machinery; however, some of these components did not reach the cut-off limit (Fig. 2, Table 1). All three detected paralogues of the scaffold protein IscA-2 and four detected copies of Nfu scaffolds were significantly upregulated; however, the expression of a single copy of IscU, which is believed to act as a principal scaffold, did not show iron-dependent regulation (Table 1). This may suggest that *Trichomonas* uses the alternative scaffolds IscA and Nfu preferentially over IscU. Unlike mitochondria, which possess two types of IscA homologues (Isa1 and Isa2), only IscA-2-encoding genes were identified in the *T. vaginalis* genome [19]. Mitochondrial Isa1 and Isa2, together with Iba57, are specifically required for the maturation of aconitase and activation of SAM enzymes [30]. In hydrogenosomes, no aconitase is present; nevertheless, the SAM enzyme HydE is essential for hydrogenase maturation ([28] see below). Therefore, one additional anticipated function of hydrogenosomal IscA-2 might be the activation of HydE. Another protein that most likely fulfills the function of a scaffold is P-loop NTPase Ind1, which is specifically required for the assembly of respiratory complex I in mitochondria [32]. Four homologues of Ind1 were detected in the hydrogenosomal proteome, which correlates with the presence of a highly reduced (two subunit) form of complex I within the organelle [10], [33]. One of the Ind1 proteins was significantly upregulated under −Fe conditions. Similarly, a single homologue of Isd11, an accessory protein of cysteine desulfurase (IscS) [34], was significantly upregulated under −Fe conditions, while the upregulation of the second detected copy of this protein and IscS itself was not significant. Consistent with the previous study of Sutak et al. [28], our study detected only IscS-2, and IscS-1 was not found, suggesting that only one of the two gene copies is expressed. Of the components that act late in FeS protein biogenesis and ensure the transfer of the nascent FeS cluster to the target apoprotein, two homologues of the chaperone HSP70 and one nucleotide exchange factor Mge1 (GrpE) were significantly upregulated under -Fe conditions.

Hydrogenosomal Hyd machinery, which consists of the three proteins HydE, HydF and HydG, is essential for maturation of the H cluster, the active site of [FeFe] hydrogenase [31], [35]. Interestingly, one paralogue of each component was upregulated under –Fe conditions, and moreover, HydG-1 showed the highest fold change between the two iron conditions (Table 1).

The observed upregulation of virtually all ISC components under iron limitation suggests the existence of a common regulatory mechanism. Multifarious Myb-like regulatory machinery has been shown to regulate the iron-dependent expression of hydrogenosomal malic enzyme; one of the identified myb regulatory elements was named MRE2f [16], [17], [36]. In our recent study, we found the MRE2f motif in the 5′untranslated regions of IscS-2 (TVAG_239660) and IscA2 (TVAG_456770) [18], suggesting that these genes might be regulated through the Myb system. However, the mode of regulation of the remaining ISC components remains unclear.

### Energy Metabolism

**Figure 3:**
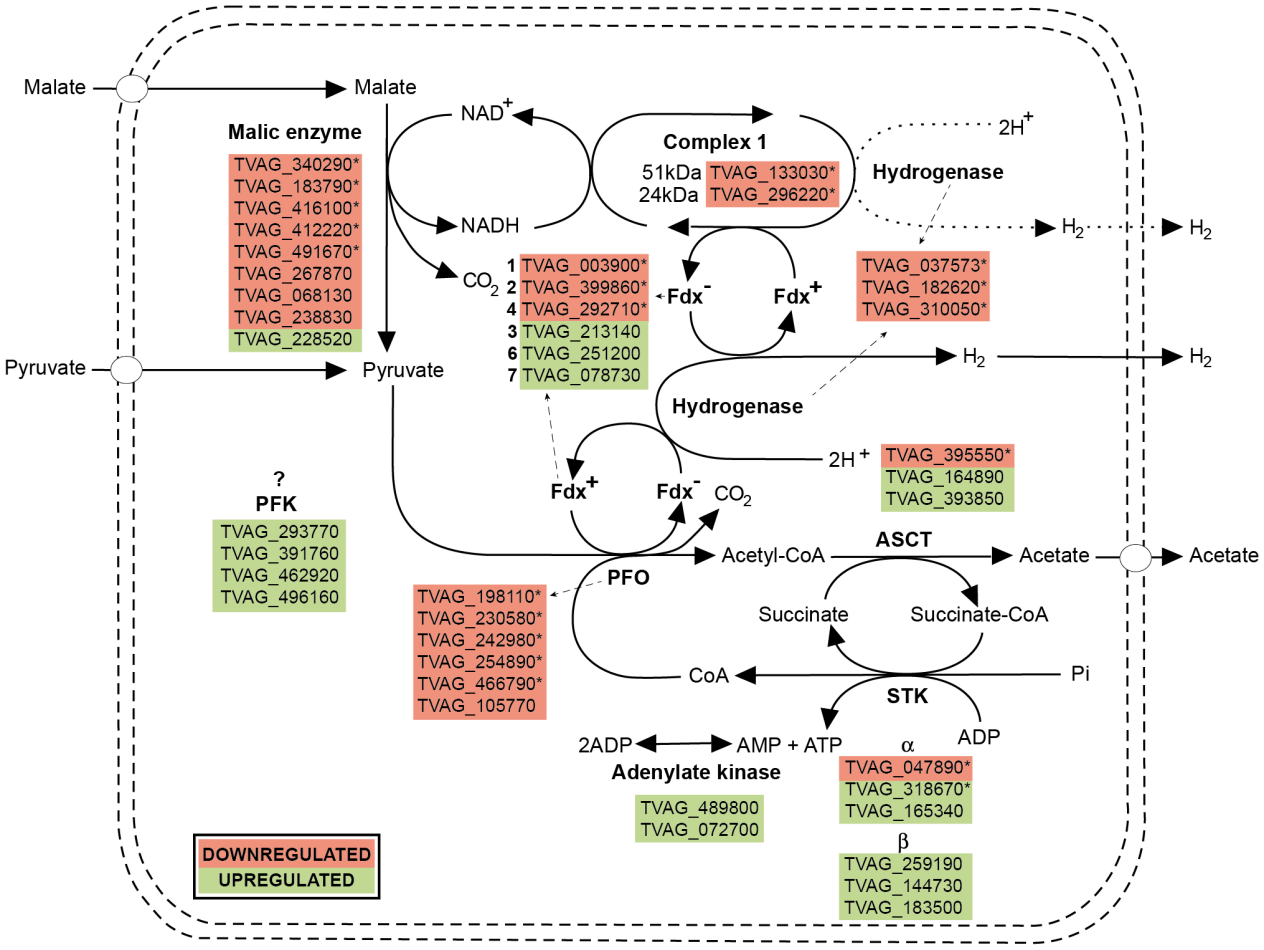
Proteins of hydrogenosomal energy metabolism identified in this study. Gene IDs marked with an asterisk denote proteins that were significantly regulated in response to iron availability.

Iron restriction resulted in the decreased expression of all enzymes involved in hydrogenosomal carbohydrate metabolism. These enzymes are encoded by multiple genes in *T. vaginalis*. Our proteomic analysis detected virtually all gene products (Fig. 3, Table 1); however, only one or several paralogues of a particular enzyme were significantly downregulated (Table 2). Two crucial FeS enzymes in the pathway, PFO and [FeFe]-hydrogenase, as well as the non-FeS malic enzyme, were among the most downregulated proteins. Three paralogues of PFO displayed downregulation one order of magnitude higher than all of the other proteins participating in energy metabolism (fold changes of −37.4, −16.9 and −13.5 for PFO BI, BII and E, respectively, Table 1). Only one of the detected paralogues was significantly downregulated in the following cases: both subunits of the respiratory complex I remnant, acetate:succinate-CoA transferase (ASCT) and heterodimeric succinyl-CoA synthase (SCS), of which only the α subunit showed significant downregulation under -Fe conditions.

Taken at face value, it appears that the above-described changes in the expression of proteins involved in iron-sulfur cluster assembly and energy metabolism are adaptive and aimed at minimizing the negative effects of iron shortage. While the pathway of hydrogenosomal pyruvate catabolism, in which abundant FeS proteins operate, is downregulated to lower the need for iron supply, the ISC system is strengthened, apparently to increase the efficiency of FeS cluster formation, which is needed for vital housekeeping proteins.

### Oxygen Detoxification System

Although *T. vaginalis* inhabits oxygen-poor environments, it is well adapted to survive periods of relative aerobiosis. The parasite possesses a number of defense mechanisms that provide protection against oxidative damage to vulnerable cellular components, among which the hydrogenosomal enzymes PFO and hydrogenase are particularly oxygen sensitive. The spectrum of proteins that are able to play a role in hydrogenosomal oxygen and reactive oxygen species (ROS) detoxification seems to be unexpectedly broad [34], [11], [9], [14]. We detected several proteins that are possibly involved in oxygen and ROS defense whose expression was influenced by iron availability. Two thioredoxins and one protein with similarity to bacterial OsmC proteins were upregulated, while rubrerythrin and superoxide dismutase were downregulated under -Fe conditions (Table 1). Thioredoxins and thioredoxin peroxidases are components of the ubiquitous peroxiredoxin system, which provides protection against peroxides similar to bacterial-type OsmC proteins, which are believed to act upon organic hydroperoxides [37], [38] (the function of the OsmC homologue in *T. vaginalis* is nevertheless unknown). It is noteworthy that while the above three upregulated proteins are non-Fe enzymes whose activity is based on cysteine residues, rubrerythrin and superoxide dismutase, which are downregulated under iron limitation, are both Fe-containing (but not FeS cluster-containing) proteins.

In addition, one of the two detected paralogues of bacterial-type FeS flavoproteins, which are likely involved in oxygen and hydrogen peroxide detoxification, was also downregulated (Table 1). In contrast, flavodiiron oxygen reductase, the terminal oxidase of the hydrogenosome [12], was not affected under -Fe conditions.

### The Differential Regulation of Paralogous Gene Expression

**Figure 4:**
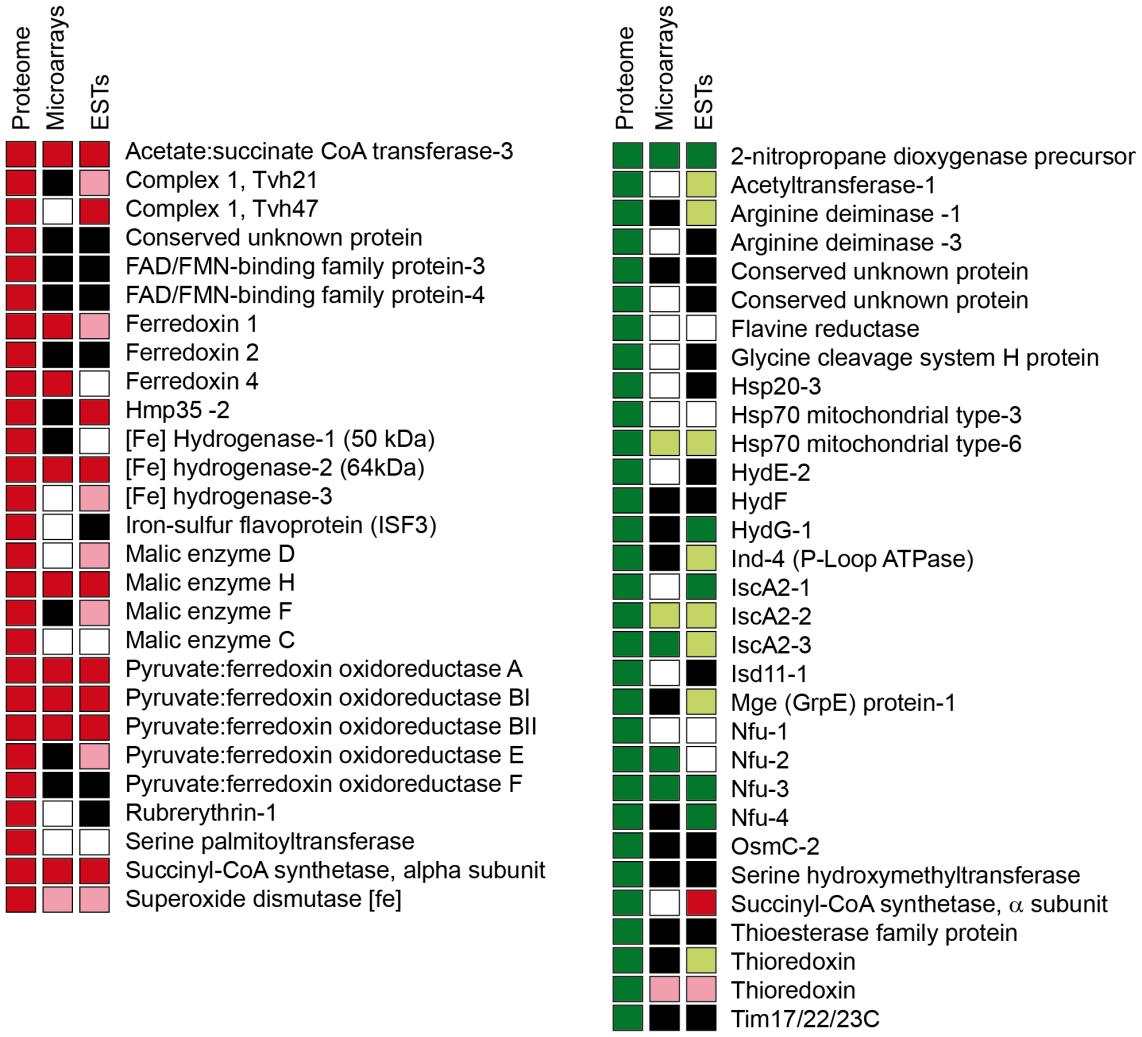
Comparison between iron-regulated proteins determined with the proteomic approach and the expression of corresponding genes studied by DNA microarrays and comparative EST analysis [**18**] **.** Red square, significant upregulation under high iron; pink square, insignificant upregulation under high iron; green square; significant upregulation under low iron; light green square, insignificant upregulation under low iron; empty square, no change in the transcript level; black square, a gene that was not included in the analysis.

The iron-dependent changes in the level of hydrogenosomal proteins determined in this study corresponded well with previous transcriptomic investigations of iron-dependent gene expression based on DNA microarray (TvArray V2.0) and comparative EST analyses (Fig. 4) [18]. The only exception observed was the α-SCS subunit (TVAG 318670), which was significantly upregulated at the proteomic level but downregulated with the EST approach under -Fe conditions. Importantly, the proteomic analysis extended the list of iron-regulated hydrogenosomal proteins, as a limited set of hydrogenosomal protein-coding transcripts was detected by DNA microarray and/or identified among *T. vaginalis* ESTs [18]. The proteomic analysis also confirmed and further elaborated a previous striking observation [18]; i.e., the differential regulation of individual copies of multi-member gene families, with some paralogues being regulated in response to changed iron concentration and others unaffected. For example, seven distinct paralogues of [2Fe-2S] ferredoxin ensure electron transport in *T. vaginalis* hydrogenosomes. Three of the paralogues (ferredoxin 1, 2 and 4) showed significant downregulation under -Fe conditions, while three paralogues (ferredoxin 3, 6, and 7) appeared to be upregulated, although their fold changes did not reach the cut-off limit (Table S1). It could be speculated that ferredoxins share the trend of regulation with the pathway in which they are involved. Therefore, we suggest that downregulated ferredoxin paralogues are involved in energy metabolism, while those that are upregulated may participate in ISC assembly. For the hydrogenase maturase HydG, the HydG-1 paralogue displayed high upregulation, with a fold change of 10.4, while the other paralogue HydG-2 showed insignificant downregulation, with a fold change of -1.6 under -Fe conditions. The observed difference in the expression of HydG paralogues may reflect their different functions or requirement for different environmental signals. Indeed, the differential expression of individual genes of multigene families was reported for *T. vaginalis* upon interaction with fibronectin [39]. For example, there are seven paralogues of thioredoxin peroxidases in the *T. vaginalis* genome [19]; of these, a single paralogue, TVAG_484570, was shown to be upregulated in parasites bound to fibronectin [39], while two different paralogues (TVAG_038090 and TVAG_455310) were significantly upregulated in cells grown under -Fe conditions (Table S1). Previous genome analyses revealed that the majority of *T. vaginalis* genes, including those coding for hydrogenosomal proteins, are present in multiple copies [13], [14], [19]. Although the reason for expansion of the *T. vaginalis* genome is not clear, it is tempting to speculate that gene multiplication together with an ability of the parasite to regulate individual genes upon different environmental stimuli provided an advantage to the parasite in its efficient response to continuous challenges in the host environment caused by factors such as the immune system, physiological changes during the menstrual cycle, iron availability and adverse microbial community.

In conclusion, the combination of transcriptomic [18] and proteomic data from trichomonads cultivated under different iron conditions presented in this work provides a basis for the study of the iron-dependent expression of individual genes that belong to multigene families, which apparently plays an important role in *T. vaginalis* cells living in rapidly changing environments.

## Supporting Information

Figure S1 **Enzymatic activities of hydrogenosomal marker malic enzyme and lysosomal marker acid phosphatase in subcellular fractions from Optiprep density gradient from iron rich and iron depleted culture.**
(TIF)

Table S1 **Complete list of proteins identified in the hydrogenosomes of Trichomonas vaginalis including likely contaminants, putative membrane proteins and hypothetical proteins.**
(XLS)

Table S2 **Bona fide hydrogenosomal proteins including putative membrane proteins.**
(XLS)
