# Supplementary figures and images for: Iron-Induced Changes in the Proteome of Trichomonas vaginalis Hydrogenosomes
Source: PLoS One. 2013 May 31;8(5):e65148. doi: 10.1371/journal.pone.0065148 (PMC3669245; doi:10.1371/journal.pone.0065148)

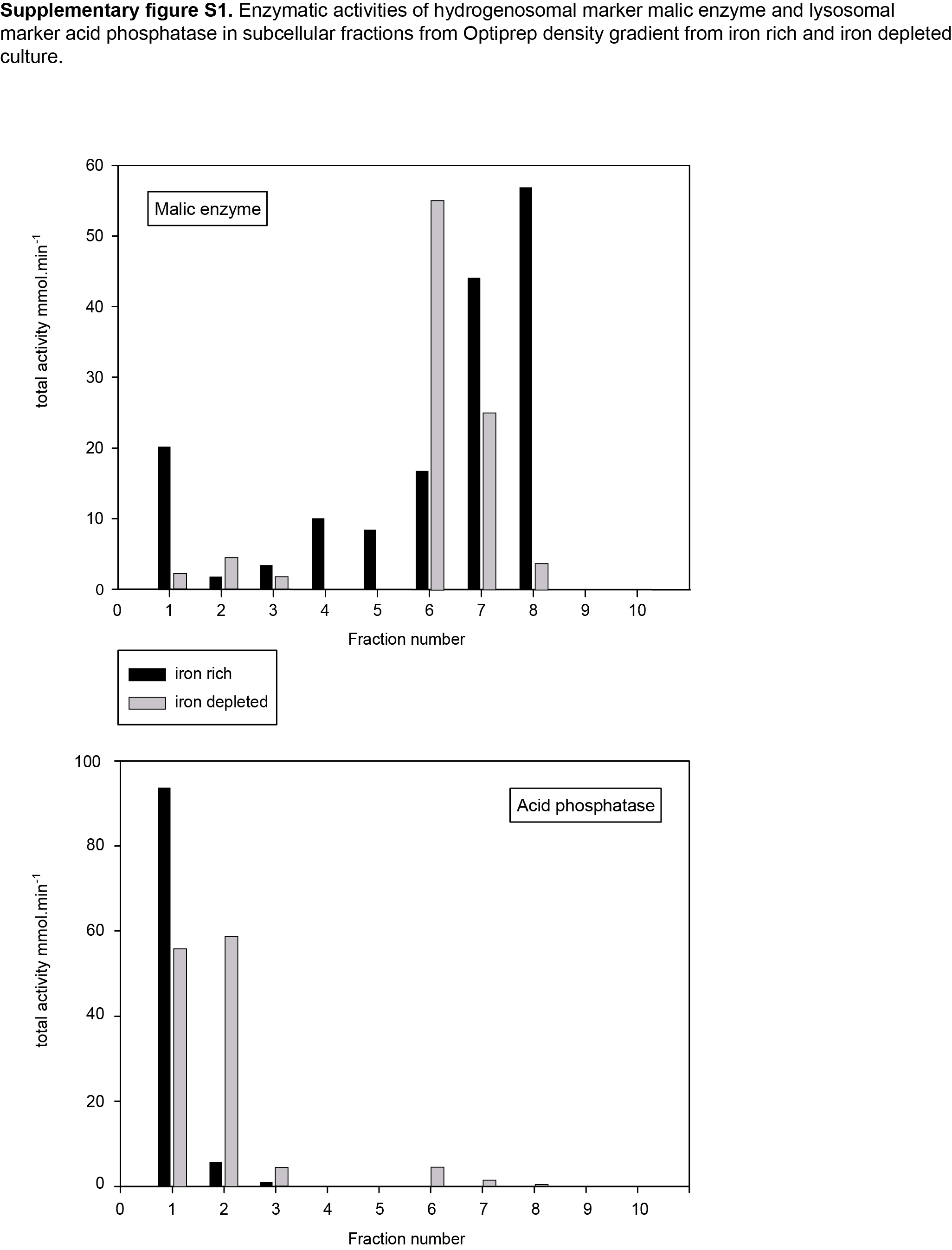

Supplement: Figure S1 — Enzymatic activities of hydrogenosomal marker malic enzyme and lysosomal marker acid phosphatase in subcellular fractions from Optiprep density gradient from iron rich and iron depleted culture. (TIF) [file pone.0065148.s001.tif]
